# Draft Genome Sequences of Enterococcus mundtii Strains Isolated from Beef Slaughterhouses in Kenya

**DOI:** 10.1128/genomeA.00446-18

**Published:** 2018-05-24

**Authors:** Joseph Wambui, Marc Stevens, Patrick Murigu Kamau Njage, Daniel Wüthrich, Adrian Egli, Roger Stephan, Taurai Tasara

**Affiliations:** a Institute for Food Safety and Hygiene, Vetsuisse Faculty, University of Zurich, Zurich, Switzerland; b Department of Food Science, Nutrition and Technology, University of Nairobi, Nairobi, Kenya; c Division for Epidemiology and Microbial Genomics, National Food Institute, Technical University of Denmark, Kongens Lyngby, Denmark; d Division of Clinical Microbiology, University Hospital Basel, Basel, Switzerland; e Applied Microbiology Research, Department of Biomedicine, University of Basel, Basel, Switzerland

## Abstract

We present here draft genome sequences of Enterococcus mundtii strains K7-EM, P2-EM, C11-EM, and H18-EM, which were isolated from slaughterhouse equipment, carcasses, and personnel of small- and medium-sized beef slaughterhouses in Kenya.

## GENOME ANNOUNCEMENT

Enterococcus mundtii strains are bacteriocin-producing enterococci that occur in natural environments, humans, and various animal species (1, 2). We report here the draft genome sequences determined for E. mundtii strains K7-EM, P2-EM, C11-EM, and H18-EM, which were isolated from equipment, personnel, and carcasses sampled in small- and medium-sized beef slaughterhouses in Kenya.

Genomic DNA isolated from the E. mundtii strains was sequenced on the MiSeq platform (Illumina, San Diego, CA, USA). The resulting genome sequences were assembled *de novo* using SPAdes genome assembler version 3.11 (3) and annotated using the NCBI Prokaryotic Genome Annotation Pipeline (4). The draft genome sequences determined in the four strains are between 3.12 Mb and 3.23 Mb in size with GC contents of 37%. Overall, there were 2,991, 3,023, 2,901, and 3,052 genes and 2,927, 2,975, 2,834, and 3,004 protein-coding sequences identified in the K7-EM, P2-EM, H18-EM, and C11-EM strains, respectively.

The numbers of RNAs predicted using the Rapid Annotations using Subsystems Technology (RAST) server (http://rast.nmpdr.org) were 62, 44, 60, and 58, while those for tRNAs predicted using tRNAscan-SE version 2.0 (5) were 53, 35, 55, and 49 in strains K7-EM, P2-EM, H18-EM, and C11-EM, respectively. In each strain, the presence of one transfer-messenger RNA was predicted using ARAGORN version 1.2.38 (6). At least four multidrug efflux pump proteins were identified in each strain using the RAST server. The macrolide resistance determinant, *ermB*, was found in strains P2-EM and C11-EM using ResFinder version 3.0 (7).

No virulence factors or phages were detected in any of the strains using VirulenceFinder version 1.5 and PHASTER, respectively (8, 9). However, the four putative hemolysin genes (hemolysin, hemolysin III, hemolysin A, and α-hemolysin), which were previously identified in E. mundtii QU 25 (10), were identified in all four strains using BLAST searches (https://blast.ncbi.nlm.nih.gov/Blast.cgi). Clustered regularly interspaced short palindromic repeats (CRISPRs) were identified using CRISPRfinder (11). H18-EM had one confirmed CRISPR, which was linked to the CRISPR-associated (*cas*) genes *cas1*, *cas2*, *cas4*, *cas9*, and *csn2*, classifying this array as a type II-A system (12). The other three strains were predicted to have between one and three unconfirmed CRISPRs.

Gene clusters encoding the production of secondary metabolites were predicted using the antiSMASH version 4.1.0 server (13). Two bacteriocin production gene clusters were detected in P2-EM and C11-EM, whereas no confirmed bacteriocin production gene cluster was identified in K7-EM or H18-EM. Limitations of the databases could have resulted in unknown bacteriocin production genes remaining unidentified. It is possible that strains K7-EM and H18-EM contain further novel bacteriocin production genes, given that *munA*, *munP*, and *munL* genes were identified in strain H18-EM using BLAST searches. *munA* is part of a gene cluster that is responsible for the production of mundticin KS (1), while *munP* and *munL* are part of a gene cluster that is responsible for the production of mundticin L (14).

### Accession number(s).

The whole-genome shotgun projects of the P2-EM, C11-EM, K7-EM, and H18-EM strains have been deposited in GenBank under the accession numbers PYGU00000000, PYGT00000000, PYGS00000000, and PYGR00000000, respectively. The versions described in this paper are the first versions, PYGU01000000, PYGT01000000, PYGS01000000, and PYGR01000000, respectively.
